# *In Vitro* Evaluation of Tellurium-Based AS101 Compound against Neisseria gonorrhoeae Infectivity

**DOI:** 10.1128/spectrum.01496-22

**Published:** 2023-03-06

**Authors:** Tsung-Ying Yang, Sung-Pin Tseng, Han-Chen Ho, Li-Hsuan Chen, Po-Ren Hsueh, Po-Liang Lu, Chia-Hsuan Lin, Liang-Chun Wang

**Affiliations:** a Department of Medical Laboratory Science, I-Shou University, Kaohsiung, Taiwan; b Research Organization for Nano and Life Innovation, Future Innovation Institute, Waseda University, Tokyo, Japan; c Research Institute for Science and Engineering, Waseda University, Tokyo, Japan; d School of Education, Waseda University, Tokyo, Japan; e Department of Marine Biotechnology and Resources, National Sun Yat-sen University, Kaohsiung, Taiwan; f Department of Medical Laboratory Science and Biotechnology, College of Health Sciences, Kaohsiung Medical University, Kaohsiung, Taiwan; g Graduate Institute of Animal Vaccine Technology, College of Veterinary Medicine, National Pingtung University of Science and Technology, Pingtung, Taiwan; h Department of Anatomy, College of Medicine, Tzu Chi University, Hualian, Taiwan; i Department of Laboratory Medicine, China Medical University Hospital, School of Medicine, China Medical University, Taichung, Taiwan; j Department of Internal Medicine, China Medical University Hospital, School of Medicine, China Medical University, Taichung, Taiwan; k Center for Liquid Biopsy and Cohort Research, Kaohsiung Medical University, Kaohsiung, Taiwan; l Division of Infectious Diseases, Department of Internal Medicine, Kaohsiung Medical University Hospital, Kaohsiung, Taiwan; m College of Medicine, Kaohsiung Medical University, Kaohsiung, Taiwan; Griffith University

**Keywords:** *Neisseria gonorrhoeae*, AS101, antibiotic resistance, biofilm

## Abstract

Neisseria gonorrhoeae (GC) is a obligate human pathogen responsible for gonorrhea, one of the most common sexually transmitted infections. The yearly increased multidrug resistance in GC has led to treatment failure clinically, suggesting an urgent need for novel therapy to combat this global health issue. AS101 [ammonium trichloro(dioxoethylene-O,O′-)tellurate], a tellurium-based compound previously used as an immunomodulatory agent, was found to have antimicrobial effects against Klebsiella pneumoniae via a high-throughput drug screening and showed antibacterial activity against Acinetobacter spp. This study aimed to evaluate the *in vitro* anti-gonococcal activity of AS101, including its antimicrobial activity, biofilm and infectivity inhibition, and potential underlying mechanisms. The agar-dilution-based MIC was used. The inhibition of GC microcolony formation and continual growth by AS101 was assessed by microscopy. The effect of AS101 on GC infectivity was evaluated by infecting endocervical ME180 and colorectal T84 epithelial cell lines. The mode of action was evaluated by a time-killing curve, transmission electron microscopy (TEM), and the level of reactive oxygen species (ROS). The MICs of MS11 and WHO GC isolates were both found to be 0.05 μg/mL. The biofilm formation, continual growth, and infectivity of two epithelial cell lines were significantly decreased with AS101 treatment. The time-kill curve, similar to that of azithromycin, suggested that AS101 is a bacteriostatic antimicrobial. However, TEM and ROS levels implied a mode of action different from that of azithromycin. Our findings highlighted the robust anti-gonococcal activities of AS101, which potentiates its use as a future antimicrobial for GC.

**IMPORTANCE** Neisseria gonorrhoeae is an obligate human pathogen responsible for gonorrhea, one of the most common sexually transmitted infections. The yearly increased multidrug resistance in GC has led to treatment failure clinically, suggesting an urgent need for novel therapy to combat the global health issue. This study aimed to evaluate the *in vitro* anti-gonococcal activity of a previous immunomodulatory agent, AS101, and its underlying mechanisms. Here, we report that AS101 possesses remarkable anti-gonococcal activity. These findings supported further studies on *in vivo* experiments and formulations for the clinical application of AS101 as an anti-gonococcal agent.

## INTRODUCTION

Neisseria gonorrhoeae (GC) is the obligate human pathogen responsible for gonorrhea, the second most common sexually transmitted diseases (STD) worldwide. The World Health Organization reports a global estimate of 87 million new gonorrhea cases annually (WHO) (1); in particular, increasing instances of infections in people between 15 and 44 years old (2) (mostly sex workers [3]). Common symptoms of gonorrhea include painful urination, genital pain, and abnormal discharge (3), and females can develop severe complications, including pelvic inflammatory disease, disseminated gonococcal infection, ectopic pregnancy, and infertility (4). The asymptomatic infection rate may be up to 56% in men and 80% in women (5), leading to the underestimation of cases, untraceable transmission, and infection control obstacles. Furthermore, GC-induced co-infection with HIV has also been reported (6), causing a considerable burden on the medical system. GC infections have been documented as the interaction with reproductive epithelium and colonization by biofilm formation (7). The interaction between GC and the epithelium involves adherence to the host epithelium, invasion of epithelial cells, and transmigration into deeper tissues for further infections or persistence. In biofilm formation, the other feature of GC infections, it has been reported that microcolonies may help the survival of GC under ceftriaxone treatment (8). In light of these characteristics, bactericidal activity against GC and the reduction or inhibition of GC-epithelial interaction and biofilm formation are considered the indices for evaluating anti-gonococcal agents.

Due to the high prevalence of quinolone-resistant GC reported (9), cephalosporins and azithromycin have been recommended to treat gonococcal infections (10, 11). Increased antibiotic consumption has been correlated with the increased antibiotic resistance of GC reported in 2016 (1), especially for the extended-spectrum cephalosporins (up to 6% to 30% in the Asian-Pacific region) and azithromycin (up to 6% to 30% in China, Russia, Canada, and Oceania). More recently, high rates of ceftriaxone and azithromycin resistance in GC have also been reported in studies conducted in the United Kingdom, Japan, and China (12–14). Accordingly, the increasing emergence of drug-resistant GC has demonstrated the urgent need for novel agents against gonorrhea. However, time and economic costs limit the development of novel agents. As an alternative, drug repurposing has been reported as an attractive option (15).

Drugs and small compounds selected using high-throughput screening systems were employed for activity-based drug discovery. Of the approximately 3,000 drugs we screened, AS101 was the first to demonstrate remarkable antibacterial activity against Klebsiella pneumoniae (16). AS101, ammonium trichloro(dioxoethylene-O,O′-)tellurate, is a low-molecular weight (312 Da) organotellurium compound (17) for which the 50% cytotoxic concentration (CC_50_) in Vero cells is 145 mg/L (18). AS101 has been applied as an immunomodulatory agent to treat tumors, autoimmune diseases, and human papillomavirus (19, 20). Furthermore, phase I/II clinical trials of AS101 as a treatment for neovascular age-related macular degeneration have been completed (NCT03216538). With unexpected antimicrobial activities, AS101 also exhibited treatment effects on bacterial, parasite, and virus infections in mouse models (16, 18, 21–24). Encouraged by these previous efforts, we sought to investigate the *in vitro* anti-gonococcal activity of AS101 through its effects on the infection ability, microcolony formation, and biofilm dissemination of GC.

## RESULTS

### MIC of AS101.

MS11, its invasive variant MS11Δ*opa* (25), and azithromycin-resistant WHO Y and Z strains were tested for the AS101 MIC via the agar dilution method. All isolates tested in this study showed the same MIC value of 0.05 μg/mL (Table 1), which is far below the CC_50_ of 145 μg/mL AS101 reported in epithelial cells (18). The susceptibility testing results indicated that AS101 could potentially serve as an anti-gonococcal agent.

**TABLE 1:** MICs of AS101 in MS11 and WHO strains^*a*^

Strain	AS101 MIC (μg/mL)							
	0	0.01	0.025	0.05	0.1	0.25	0.5	1
MS11WT	+	+	+	−	−	−	−	−
MS11Δ*opa*	+	+	+	−	−	−	−	−
WHO Y	+	+	+	−	−	−	−	−
WHO Z	+	+	+	−	−	−	−	−

a MS11WT, MS11Δ*opa*, WHO Y, and WHO Z were grown on GCK agar and suspended in GCP. Agar dilution test was then performed using GCK plates with concentrations of AS101 from 0.001 to 1.0 μg/mL.

### Time-kill curves of AS101.

**FIG 1:**
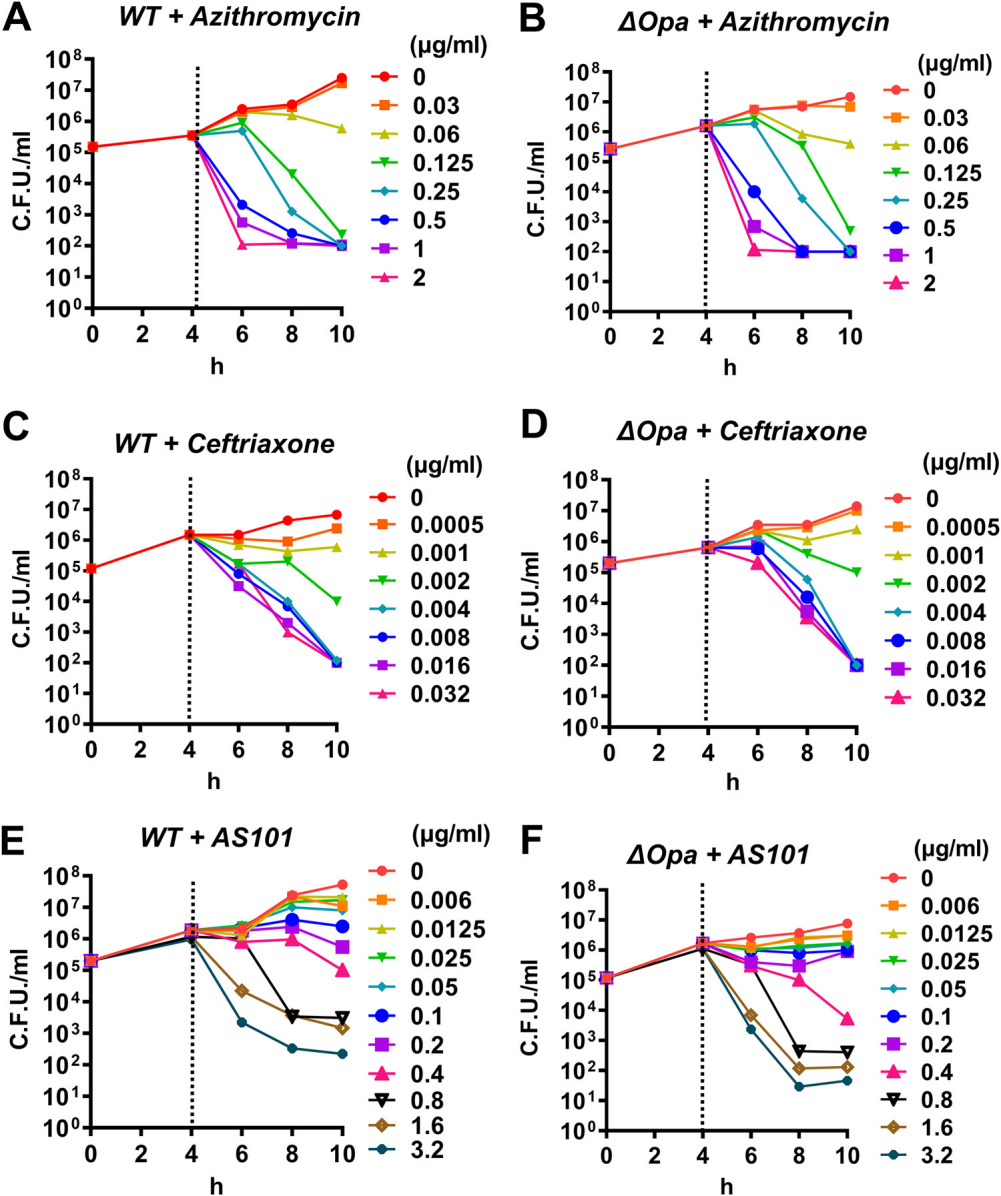
Time-kill curves for Neisseria gonorrhoeae GC using AS101 [ammonium trichloro(dioxoethylene-O,O′-)tellurate] and two different antibiotics. MS11WT (WT, wild-type) and Δ*opa* were seeded into well plates and incubated for 4 h. (A and B) Azithromycin, (C and D) ceftriaxone, or (E and F) AS101 was added to the well. Here, 8 to 11 doubling dilutions are plotted in different colors, and growth in the absence of antimicrobial is plotted in red. The antimicrobial was added at 4 h (dash line) and growth was monitored until 10 h. The limit of detection in this assay was 100 CFU/mL. Dots represent the means for each group, and bars indicate standard deviations. All experiments were conducted in triplicate.

Because all strains described above had the same MIC, we used MS11WT (WT, wild-type) and MS11Δ*opa* for the rest of the antimicrobial tests to better compare our results with previously obtained infectivity information. AS101, azithromycin, or ceftriaxone was added into a 4-h incubated GC suspension and growth was continuously observed for 6 h by agar plating every 2 h. The time-kill curves of azithromycin (Fig. 1A and B) and ceftriaxone (Fig. 1C and D) for both strains showed a similar and significant decrease in growth after 6 h of incubation at concentrations of 0.06 and 0.002 μg/mL, respectively. Following its bacteriostatic and bactericidal properties, the time-kill curve of azithromycin showed a gradual decrease of growth in a time-dependent manner, whereas ceftriaxone showed a sharp decrease in growth after 0.002 μg/mL. The time-kill curve for both MS11WT and MS11Δ*opa* showed a gradual decrease in growth as the AS101 concentration increased (Fig. 1E and F). Accordingly, significant decreases in growth were found at 0.04 and 0.08 μg/mL at 6 and 8 h, respectively. These data imply the possible bacteriostatic property of AS101 in inhibiting GC growth.

### AS101 reduces the formation and growth of GC microcolonies.

**FIG 2:**
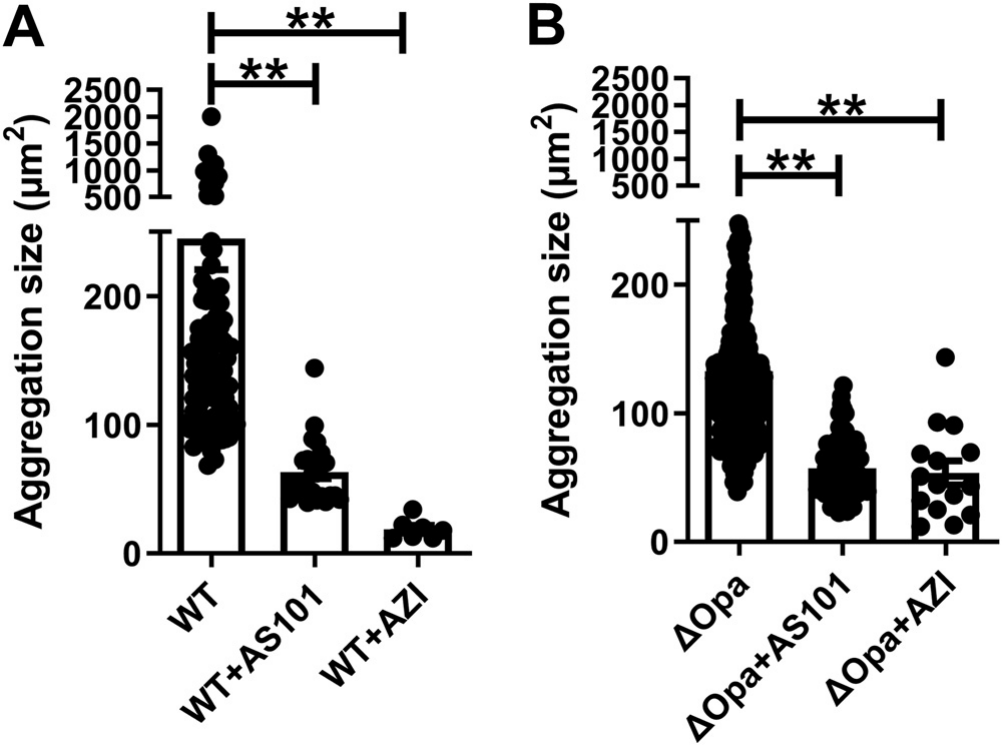
Effects of AS101 on GC microcolony formation. (A) MS11WT and (B) MS11Δ*opa* at 2 × 10^6^ cells were seeded in a coverslip-bottomed chamber in the presence or absence of AS101 or azithromycin, incubated for 4 h, and imaged using a Zeiss Axio Observer microscope. Aggregation sizes were evaluated by measuring the average occupying area of individual aggregates in each image using NIH ImageJ. Data were generated from eight randomly acquired fields from four independent experiments. Each data point indicates an individual aggregation. Bars represent means of each group; lines on bars indicate standard deviations. Statistical significance was determined using one-way analysis of variance (ANOVA) followed by Tukey’s *post hoc* tests (***, *P* < 0.001; **, *P* < 0.01; *, *P* < 0.05).

Previous studies have reported that GC can form microcolonies and later develop into a biofilm. Therefore, we sought to evaluate the effect of AS101 on microcolony formation. MS11WT and MS11Δ*opa* were inoculated into an 8-well chamber slide in the absence or presence of 1 μg/mL AS101 or 0.5 μg/mL azithromycin for 4 h. Microcolony formation was determined by analyzing the size of the aggregation imaged using a light microscope. We found that the average aggregation size of MS11WT (Fig. 2A) was larger than that of MS11Δ*opa* (Fig. 2B); as expected, the aggregation sizes of both were significantly reduced with AS101, similar to azithromycin, in comparison with the non-treatment control in both strains (Fig. 2A and B). The aggregation property of MS11 in AS101 and azithromycin was similar to that of MS11pili-strain, which shows little to no aggregation (8). This result suggests that AS101 can inhibit microcolony formation. We then hypothesized that the inhibition of microcolony formation results from reduced cell viability and/or related cellular processes based on the time-kill curve. To examine GC survival within the aggregation, Live/Dead staining was applied to MS11Δ*opa* aggregates incubated with or without AS101 or ceftriaxone as a control. Next, 3D confocal microscopy was used to visualize distribution of live and dead bacteria and the size of GC aggregates. We observed a gradual loss of live bacterial cells and aggregation size with increasing concentrations of AS101 compared to the non-treatment control (Fig. S1A in the supplemental material). We then measured the aggregation size and fold change of the live/dead ratio from the images for quantitative analysis. A significant 1.4- to 24-fold reduction in aggregation size was found for the group treated with 0.01 to 1 μg/mL AS101 compared to the non-treatment control (Fig. S1B). The amount of live bacteria was determined via the fluorescence intensity ratio (FIR) of live to dead bacteria in GC aggregates. We observed a 1.2- to 3.3-fold reduction in live bacteria within the aggregates from 0.01 to 1 μg/mL AS101 compared to the non-treatment control (Fig. S1C). We found that 1 μg/mL AS101-treated GC formed GC aggregates with similar sizes and live bacteria levels to the ceftriaxone control. These observations demonstrated that AS101 can inhibit the formation of GC aggregation by reducing bacterial viability.

**FIG 3:**
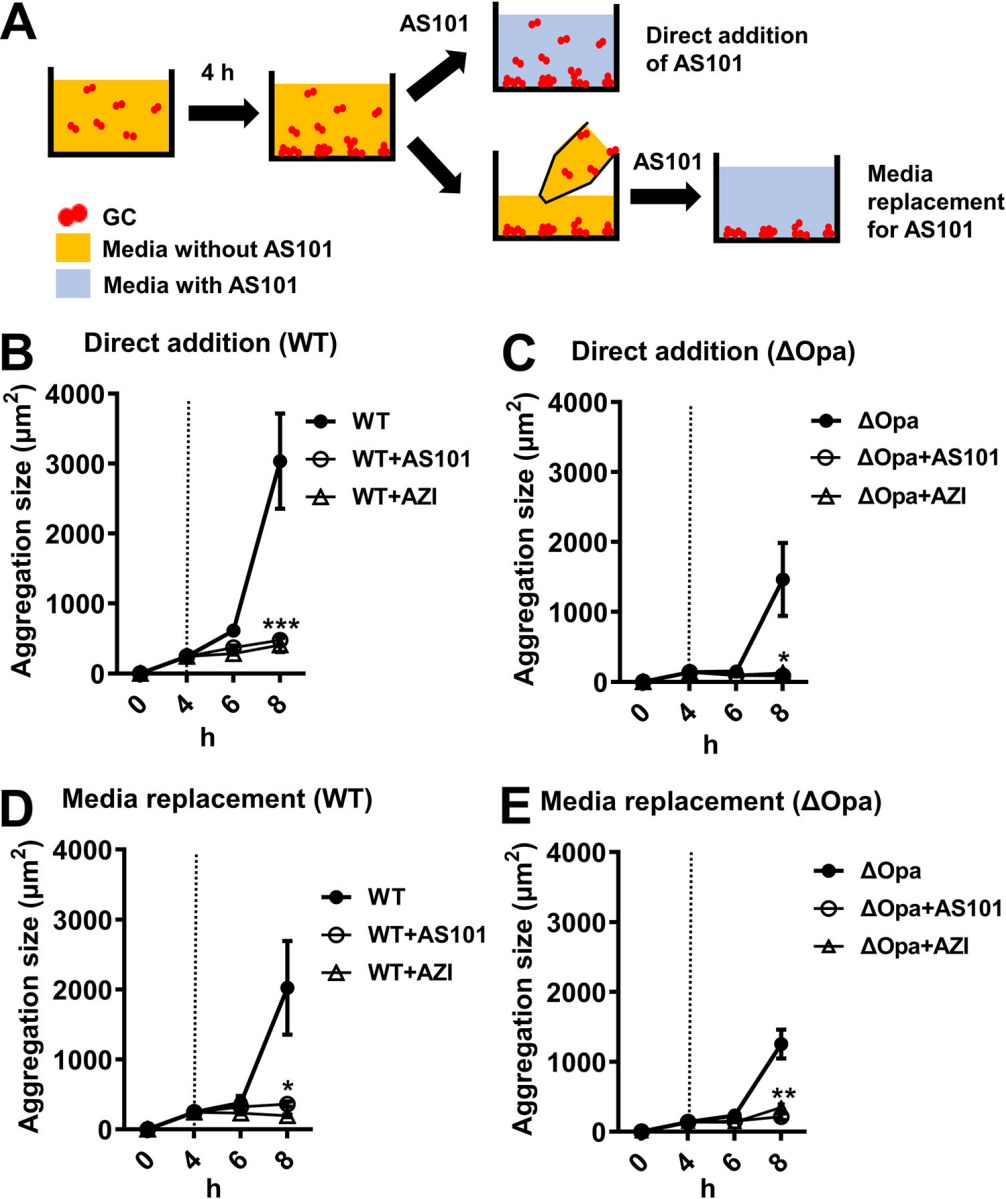
Effects of AS101 on existing GC microcolonies. (A) Illustration of experiment. (B and C) MS11WT and (D and E) MS11Δ*opa* were seeded in a coverslip-bottomed chamber and incubated for 4 h. AS101 or azithromycin was then (B and D) directly added into the chamber or (C and E) added to the medium to replace the GC suspension (dashed line). Aggregation size was determined using a Zeiss Axio Observer microscope at 2 and 4 h post-treatment. Aggregation sizes were evaluated by measuring the average occupying area of individual aggregates in each image using NIH ImageJ. Data were generated from eight randomly acquired fields from four independent experiments. Each data point indicates an individual aggregation. Dots represent the means of each group, and the bars on dots indicate standard deviation. Statistical significance was determined using one-way ANOVA followed by Dunnett’s *post hoc* test (***, *P* < 0.001; **, *P* < 0.01; *, *P* < 0.05).

While AS101 was shown to inhibit the formation of GC microcolonies from individual GC, whether it can inhibit the growth of existing microcolonies is unknown. To examine microcolony growth inhibition, we used the same method as above, but with the antimicrobials added after the formation of GC microcolonies. MS11WT and MS11Δ*opa* were inoculated into an 8-well chamber slide and incubated for 4 h. AS101 or azithromycin was then added into the well at the 4-h time point (Fig. 3A, upper), and microcolony growth was monitored with a light microscope every 2 h at the 6- and 8-h time points. MS11WT and MS11Δ*opa* showed a gradual increase in aggregation at 6 h, and a significant increase was observed at 8 h, indicating the growth and binding of microcolonies (Fig. 3B and C). After treatment with AS101 or azithromycin, the size of aggregation remained similar throughout the 4-h treatment. The aggregation sizes in the AS101 and azithromycin treatment groups was significantly reduced compared to non-treatment control at the 8-h time point (Fig. 3B and C). These data suggest that the growth and binding of microcolonies can be inhibited. To further confirm that the binding of each microcolony was inhibited, the inoculated medium along with suspended GC was aspirated after 4 h and replaced with fresh medium containing AS101 or azithromycin only (Fig. 3A, lower). We found that both strains were unable to increase their aggregation size with AS101 or azithromycin added, while significantly increased aggregation size was still observed in the non-treatment control, suggesting that microcolony binding was inhibited. Taken together, these data suggest that AS101 can inhibit both the formation and growth of GC microcolonies.

### AS101 inhibits GC adherence and invasion of the endocervical and anorectal epithelial cells.

**FIG 4:**
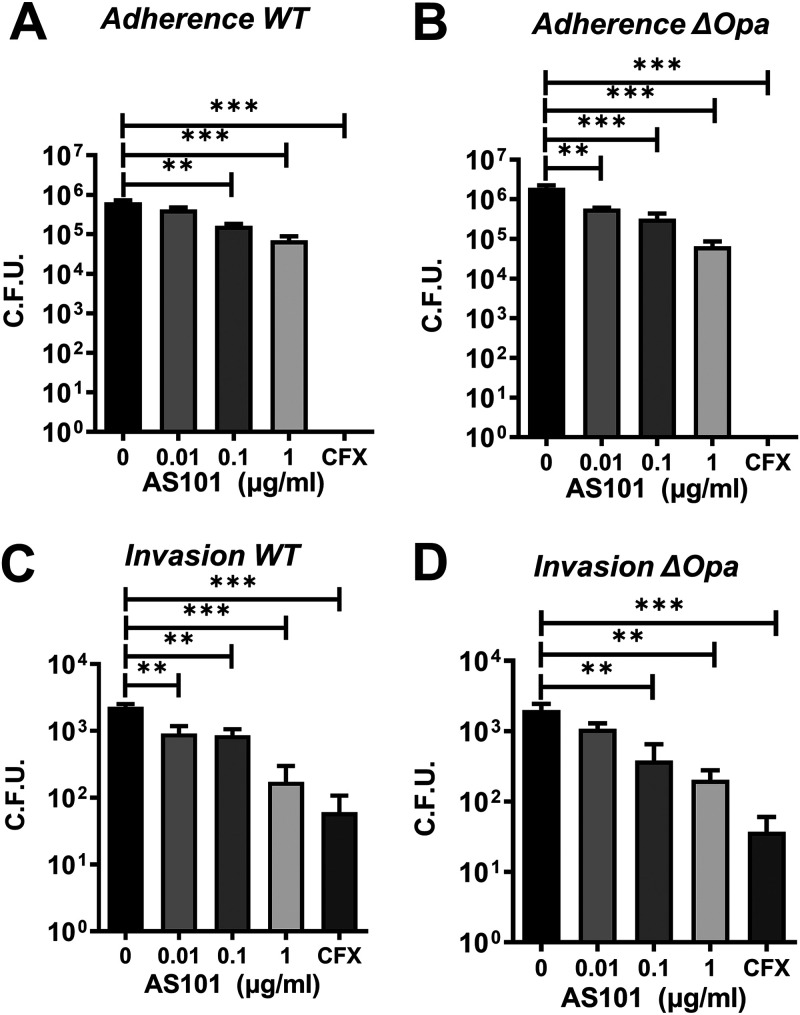
Effects of AS101 on GC infectivity. ME180 cells were grown on transwell inserts and apically inoculated with MS11WT or MS11Δ*opa* . (A and B) Various concentrations of AS101 was added apically along with GC inoculation and incubated for 3 h to determine the adherent GC. (C and D) Inoculated suspension was aspirated and replaced by various concentrations of AS101 at the 3-h time point and incubated for another 3 h. Invading GC was quantified by a gentamicin protection assay. GC treated with 1 μg/mL of ceftriaxone served as a positive control. Bars represent the means of each group, and lines on bars indicate the standard deviation. Statistical significance was determined using one-way ANOVA followed by Dunnett’s *post hoc* test (***, *P* < 0.001; **, *P* < 0.01; *, *P* < 0.05). All exepriements were conducted at least in triplicate.

GC infection has been described as adherence to epithelium, invasion into epithelial cells, and/or transmigration into subepithelial tissue (26). Adherence is the initial and critical step for determining whether GC colonizes the mucosal surface. To examine whether AS101 affects adherence, MS11WT and MS11Δ*opa* were inoculated onto a ME180 human epithelial cell layer with or without AS101 at various concentrations for 3 h. Cells were then collected for agar plating and adherent GC counting. Ceftriaxone at a final concentration of 1 μg/mL served as the positive control. We found a significant decrease in adherence up to 10-fold, starting from 0.1 and 0.01 μg/mL of AS101 in MS11WT (Fig. 4A) and MS11Δ*opa* (Fig. 4B), respectively. However, no adherent GC was observed with ceftriaxone at 1 μg/mL. These data indicate the adherence-inhibition effect of AS101 as a bacteriostatic instead of a bactericidal agent.

We then sought to examine whether AS101 can inhibit GC invasion into epithelial cells. MS11WT and MS11Δ*opa* were inoculated onto a ME180 human epithelial cell layer for 3 h to represent equal adherence. The GC suspension was then switched with medium containing various concentrations of AS101 or 1 μg/mL of ceftriaxone for another 3 h of incubation. The gentamicin-treated and saponin-lysed cells were examined to determine the invading GC. We found a significant decrease in invasion with 0.01 and 0.1 μg/mL of AS101, up to a 10-fold decrease in MS11WT (Fig. 4C) and MS11Δ*opa* (Fig. 4D), compared to ceftriaxone treatment. These data suggest that AS101 can inhibit invasion even after GC colonization. Similar findings for adherence and invasion, along with reduced transmigration, were also found when we used a polarized T84 cell monolayer to exclude cell-dependent effects (Fig. S2). These findings suggested that AS101 can inhibit GC infection, including adherence and invasion, even at a sub-MIC depending on the strain.

### Morphological change of GC under AS101 treatment.

**FIG 5:**
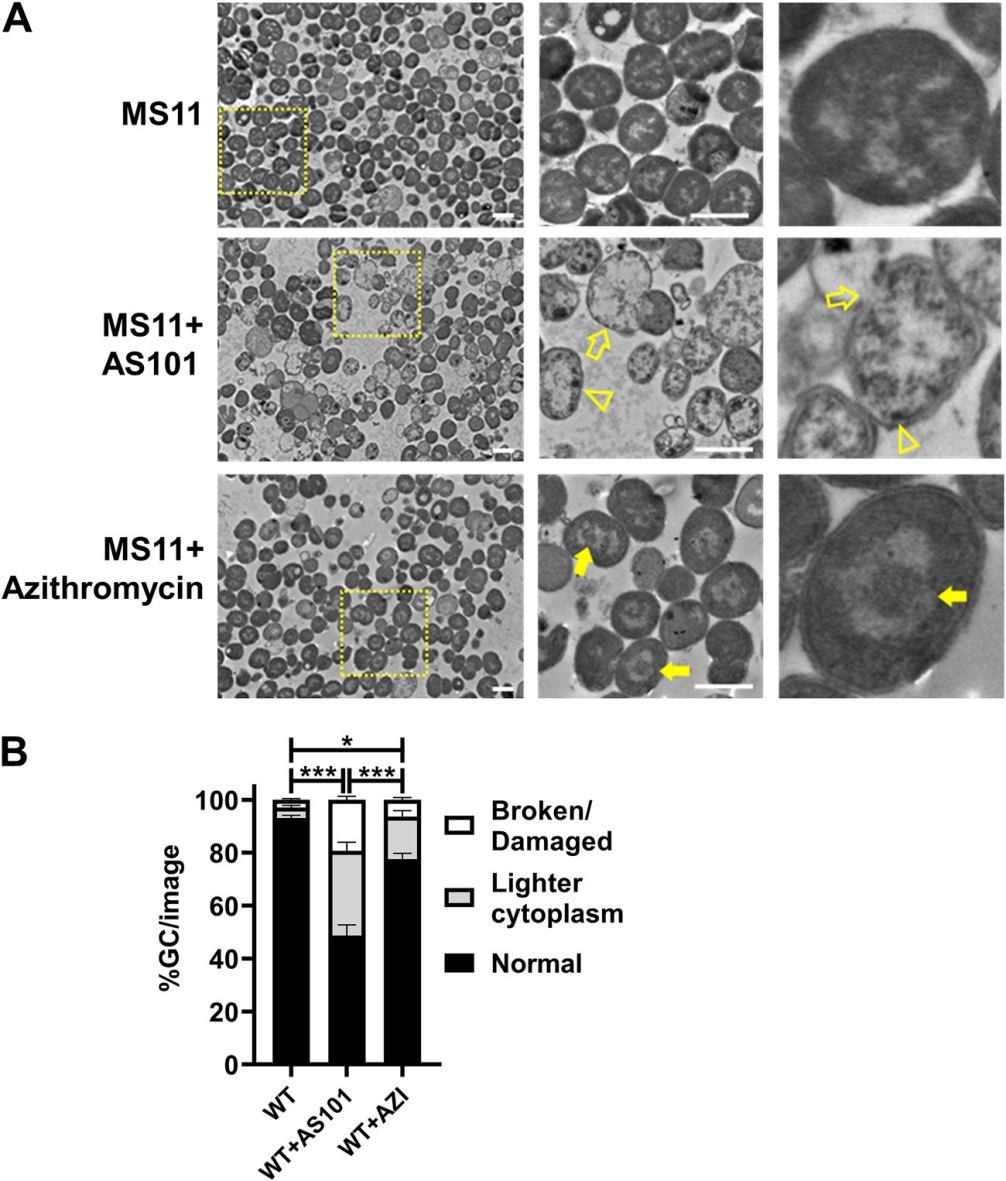
Morphological and structural impact of AS101 on GC. Here, 10^8^ freshly grown MS11WT were incubated in GCP broth in the absence or presence of AS101 (1 μg/mL) or azithromycin (0.5 μg/mL) for 4 h. Cells were fixed and processed for transmission electron microscopy (TEM). (A) Representative images at low (left panels) and high (right panels) magnification. Yellow dashed-line rectangles highlight the focused area. Open arrows, enlarged GC with cytoplasm loss. Open arrowheads, condensed deposits accumulating at the inner membrane of GC. Solid arrows, toroid nucleoids. (B) Morphological changes in cell were quantified into the percentage of GC in the non-treatment control. Data points represent individual randomly acquired 8 TEM images. Bars represent the means of each group, and lines on bars indicate the standard deviation. Scale bar = 1 μm. Statistical significance was determined using one-way ANOVA followed by Tukey’s *post hoc* tests (***, *P* < 0.001; **, *P* < 0.01; *, *P* < 0.05).

The collective evidence from these results indicates that the bacteriostatic property of AS101 may be similar to that of azithromycin. Whether AS101 exerts a similar mode of action as azithromycin is unknown. To evaluate the mode of action of AS101, MS11WT and MS11Δ*opa* were incubated with 1 μg/mL AS101 for 4 h, followed by transmission electron microscopy (TEM) preparation. The cell morphology and cytoplasmic structure were imaged and analyzed. Azithromycin at a final concentration of 0.5 μg/mL served as the positive control. We found that non-treatment MS11WT showed a typical coccus shape, double-membrane structure, and dense cytoplasmic contents (Fig. 5A, upper). Compared to the MS11 control, AS101-treated MS11WT showed enlarged cells, occasional membrane rupture, and light cytoplasmic contents with dense deposits attached to the inner membrane (Fig. 5A, middle). Azithromycin-treated MS11WT exhibited a coccus morphology, comparative cell size, and dense cytoplasmic contents similar to that of non-treated MS11WT, while a torroid nucleoid was found (Fig. 5A, lower), similar to what Lin et al. reported for Acinetobacter baumannii (27). We then categorized the cell morphology into broken/damaged, lighter cytoplasmic, and typical types for quantification The results showed that damaged and electron-lucent cells were quantitatively and significantly increased in AS101-treated GC compared to either untreated or azithromycin-treated MS11WT, implying the loss of cell contents and membrane instability following AS101 treatment. These data indicate that AS101, although considered a bacteriostatic antimicrobial agent, has a mode of action different from that of azithromycin.

### AS101 induces reactive oxygen species in GC.

**FIG 6:**
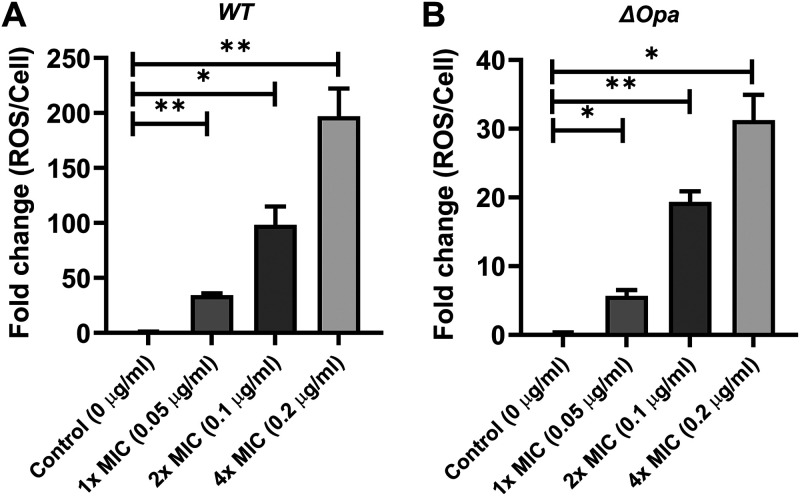
Reactive oxygen species (ROS) levels in MS11WT (A) and MS11Δ*opa* (B). A DCFH-DA (2′,7′-dichlorodihydrofluorescein diacetate) assay was utilized to determine intracellular ROS levels. In brief, 10^7^ CFU/mL of bacterial strains in GC broth were incubated with 1× MIC (0.05 μg/mL), 2× MIC (0.1 μg/mL), or 4× MIC (0.2 μg/mL) AS101 or control treatement (0 μg/mL) for 1 h at 37°C. Fluorescence signals were measured with an excitation wavelength of 500 nm and an emission wavelength of 530 nm. To normalize fluorescence intensities, viable bacteria were counted by serially diluting and plating the samples onto GC agar plates. All experiments were conducted in triplicate. Bars represent the means of each group, and lines on bars indicate the standard deviation. Statistical significance was determined using one-way ANOVA followed by Dunnett’s *post hoc* test (**, *P* < 0.01; *, *P* < 0.05). All exepriements were conducted at least in triplicate.

TEM images showed the loss of cell contents and membrane stability, indicating the short-term stress in discontinuing the cellular process. To this end, we measured intracellular ROS levels for MS11 WT and MS11Δ*opa* strains treated with AS101 for 1 h. As shown in Fig. 6A, the MS11 WT bacterial cells treated with 1×, 2×, and 4× MIC AS101 exhibited significantly higher ROS generation than the control group, where the ROS levels increased in a dose-dependent manner. Similarly, MS11Δ*opa* was found to have significant ROS increases in the groups treated with AS101 at 1×, 2×, and 4× MIC (Fig. 6B). These observations confirmed that AS101 potentially induces short-term ROS bursts as stress to inhibit the cellular process of GC.

## DISCUSSION

The expensive and time-consuming processes for drug development have impeded the launch of newly approved antibiotics; in parallel with this, antibiotic resistance in GC increases yearly, implying that drug repurposing could be an efficient and alternative option to combat this dilemma (15). Previous efforts on drug repurposing against GC have included fenamic acid (nonsteroidal anti-inflammatory drug) (28), sulfonamide (carbonic anhydrase inhibitors) (29), and salicylamide (analgesic and antipyretic drug) (30). Seong et al. (28) repurposed fenamic acid drugs as anti-gonococcal agents, with MIC_50_ values ranging from 4 to 16 μg/mL. Abutaleb et al. (29) reported the anti-gonococcal activity of ethoxzolamide, an FDA-approved human carbonic anhydrase inhibitor, which exhibited an MIC_50_ of 0.125 μg/mL. Alhashimi et al. (30) discovered that salicylamide possessed antibacterial activity against 40 antibiotic-resistant GC strains, with a MIC range of 8 to 32 μg/mL. The tellurium-containing organic compound AS101 was first used as an immunomodulatory agent to treat autoimmune diseases and tumors (19, 20), and more recently, a phase I/II clinical trial of AS101 for neovascular age-related macular degeneration was completed (NCT03216538), suggesting a potential for development. Although previous efforts have revealed that AS101 possesses antimicrobial effects against Enterobacter cloacae (23), K. pneumoniae (16), and A. baumannii (24), little is known about its anti-gonococcal activity. Yang et al. (16) reported effective antibacterial activity of AS101 against K. pneumoniae, with MICs ranging from 0.5 to 32 μg/mL. The time-kill assays showed rapid bacterial eradication for AS101, where 97.9%, 96.8%, and 96.8% of bacteria, compared to the starting inocula, were eliminated by 1×, 2×, and 4× MICs of AS101, respectively, within 1.5 h. Furthermore, AS101 also possessed a robust *in vivo* treatment effect to rescue up to 75% of sepsis mice infected by K. pneumoniae, implying a potential clinical application. Another study conducted against A. baumannii (24) produced similar results, with a MIC range of 0.5 to 32 μg/mL, 99.9% of bacterial elimination, and a survival rate of up to 58.3% in the sepsis mouse model. In the current study, we examined the susceptibility, infectivity inhibition ability, and possible mode of action of AS101 and demonstrated that it possessed remarkable anti-gonococcal activity against GC, potentially through ROS-based reduction of bacterial viability.

The pathogenesis of GC is mainly attributed to three interrelated stages: adherence, transmigration, and invasion (31). In a previous study, significant intracellular clearance (elimination of GC invasion) was observed for tolfenamic, flufenamic, and meclofenamic acids at 6× MIC (all *P* < 0.01), with approximately 2-log bacterial reductions (28). In another study, the effectiveness of salicylamide against intracellular GC was demonstrated, with a significant 2-log decrease while treating infected human endocervical cells with 5× MIC (30). The anti-infective effects of AS101 were examined using adherence, invasion, and transmigration assays. Our results suggested that the lowest concentration of AS101 with an anti-adherent effect in treating both MS11WT and MS11Δ*opa* strains was 0.1 μg/mL (Fig. 4A and B). Moreover, invasion and transmigration could be inhibited with 0.01 μg/mL of AS101 even after successful adherence had been established (Fig. 4C and D, Fig. S2). This concentration difference suggests that AS101 enters epithelial cells and damages the invading GC. Indeed, we investigated this possibility by adding AS101 while performing the gentamicin assay. A significant reduction of invading GC was observed in the gentamicin assay with AS101 compared to the gentamicin assay alone (Fig. S3), indicating that AS101 entered epithelial cells and exerted an anti-GC effect. These findings suggested that AS101 could potentially be used as an anti-GC treatment at any stage of GC infection or with different strains. It would be interesting to investigate how long AS101 can stay in its effective form in the epithelial cells prior to GC infection for the development of anti-GC prevention measures.

Biofilm formation has been reported to impede the antibacterial activity of antibiotics (32). GC biofilms begin with the formation of, and then the growth and binding of, microcolonies (8). The reduced antibiotic susceptibility of GC biofilm may lead to further treatment failure and recurrence, or even drive pathogen evolution (8). To this end, inhibiting the gonococcal microcolony could be a potential strategy to develop a highly effective agent against GC infections. Goytia et al. (33) reported that spermine could notably reduce the formation of GC biofilms, but this anti-biofilm effect was barely found for the pre-formed GC biofilm. In a previous study, Daniel-Hoffmann et al. (23) found that AS101 could inhibit biofilm formation in E. cloacae using a crystal violet staining method. In our current study, GC microcolony formation and growth were found to be significantly disrupted by AS101 (Fig. 2 and 3), indicating that it is a comparative antibiotic candidate. However, how effectively AS101 inhibits GC microcolony formation and growth on epithelial cells is not known. Because our results showed the cytosolic killing of GC in ME180 cells, it would be interesting to examine the effects of AS101 on the formation and growth of GC biofilm after GC attaches to the epithelial cells.

In a previous literature review, observations of morphological changes were utilized to speculate on different antimicrobial mechanisms (34). The antimicrobial mechanisms of silver nanoparticles were attributed to the disruption of the cell membrane, oxidization of cell components, or accumulation of reactive oxygen species. In a study of silver nanoparticles against GC, the SEM images displayed enlarged cell sizes and leakage on the cell membrane. Previous studies of AS101 revealed a wrinkled or cracked surface in micrographs of E. cloacae (23) and K. pneumoniae (16) following AS101 treatment. In our study, the TEM micrographs of AS101-treated GC illustrated enlarged cells and the loss of cytoplasmic contents, but not cracked surfaces, implying an antibacterial mechanism different from those in previous work. However, it is unknown whether this is time- or dosage-dependent in different bacterial species. Interestingly, we observed deposits on the inner membrane; a similar phenomenon has been mentioned in our previous studies (16, 24). Therefore, AS101 may enter the bacterial cell and accumulate on the inner membrane. Future work could examine the distribution of AS101 in the bacterial cell using a high-angle annular dark-field (HAADF) scanning TEM to further explore the interaction of AS101 with GC and its mode of action. Furthermore, ROS, especially hydroxyl radicals, have been reported to play a critical role in the bactericidal activities of bactericidal agents (35). In previous efforts, the ROS levels in bacterial cells treated with AS101 were observed to increase in a dose-dependent manner (16, 24). In this study, significant changes in ROS levels in MS11WT and MS11ΔOpa were observed when they were treated with various concentrations of AS101 (Fig. 6). Interestingly, the bacteriostatic activity found in the time-kill assay did not agree with previous studies, implying a different mechanism of AS101 against GC that may need further genomic and transcriptomic investigation to decipher.

Opa protein was reported to potentially determine clinical outcomes in GC infection (36). Lack of Opa protein can enhance its transmigration ability but reduce microcolony sizes (8, 25). In this study, AS101 exerted similar effects on infectivity and microcolony formation and growth in both strains and no Opa-protein-dependent effects were observed, indicating that AS101 can broadly inhibit GC strains. However, AS101-induced ROS levels in MS11Δ*opa* were lower than those in MS11WT (Fig. 6), while both strains produced similar ROS levels without AS101 treatment (data not shown). This suggests a physiological difference between these two strains. It would be interesting to determine whether this is due to a difference in the Opa protein synthesis machinery or another cellular process.

Although AS101 is considered an effective anti-GC agent, the increased resistance rate is considered high due to its interpreted bacteriostatic properties. Our screening also indicated a high survival rate at less than 1× to 2× MIC in both MS11 and WHO strains (Table S1). While resistance can be expected, AS101 may be an effective anti-GC agent against azithromycin resistance. Azithromycin is a recommended treatment for gonococcal infections and has been reported to have a high resistant proportion clinically, creating an obstacle in treating infections (10, 13). The azithromycin resistance in GC could be attributed to the upregulation of the efflux pump designated the MtrCDE system (37), which was also found to downregulate the expression level of *terC*, a tellurium-resistant gene (38), potentially increasing susceptibility to tellurium-based AS101. Thus, we hypothesized that AS101 could exert increased antimicrobial effects in MtrCDE-based azithromycin-resistant GC. The preliminary data for gradient azithromycin-selected GC have shown decreased susceptibility to AS101 (Fig. S4), while MtrCDE upregulation was also found (Fig. S5), similar to previous findings. These findings supported the idea that AS101 could potentially be an anti-azithromycin-resistant GC with *mtrCDE* overexpression. However, how *terC* is regulated and the association between the MtrCDE system and susceptibility will need to be revealed.

This study demonstrated the robust anti-gonococcal activities of AS101, including its bacteriostatic properties and its biofilm and infectivity inhibition. Our findings highlighted the potential of AS101 against N. gonorrhoeae, suggesting further studies on *in vivo* experiments and formulations for the clinical application of AS101 as an anti-gonococcal agent.

## MATERIALS AND METHODS

### Bacteria strains.

The GC strain MS11 used in this study was kindly provided by Daniel C Stein (University of Maryland, College Park, MD). MS11WT and MS11ΔOpa have been previously described (25). The WHO strains Y and Z were purchased from National Collection of Type Cultures (NCTC), London, United Kingdom. All GC strains were grown on GCK agar plate (Difco, Franklin Lakes, NJ) and 1% Kellogg’s supplement at 37°C with 5% CO_2_ for 16 to 18 h before use (39).

### AS101.

The compound AS101 was supplied by the Development Center for Biotechnology (DCB), a nonprofit organization supported by Taiwan governmental funds, dissolved in 1× PBS (phosphate-buffered saline), and stored at 4°C until use.

### MICs.

MICs of AS101 against GC were determined using the agar dilution method according to CDC protocol (40). GC suspended in GC medium were diluted to 10^7^, 10^6^, 10^5^, and 10^4^ CFU/mL; 5 μL of each suspension was dropped on a GCK agar plate containing various AS101 concentrations. The MIC was defined as the concentration which inhibited at least 90% of GC growth (40).

### Time-kill assay.

Time-kill curve analyses were performed using methods adapted from Foerster et al. (41) by culturing GC in GCP broth supplemented with 1% Kellogg’s supplement and 4.2% NaHCO_3_ in the presence of antimicrobial concentrations in doubling dilutions ranging below and above the MIC. For each strain, 30 μL of the 10^8^-GC/mL inoculum was diluted in 15 mL pre-warmed supplemented GCP, and 90 μL per well was dispersed on round-bottomed 96-well microtiter plates. To each well containing 90 μL of pre-incubated bacteria, 10 μL of one of the antimicrobial concentrations was added. The plates were pre-incubated for 4 h shaking at 550 rpm, 36°C in a humid PST-60HL Thermo-Shaker (Biosan, Riga, Latvia). Next, 10 μL of the GC suspension from each well was spotted onto a GCK plate and incubated overnight to determine the amount of viable GC.

### Epithelial cells.

Human ME180 endocervical (ATCC no. HTB-33) and T84 colonic (ATCC no. CCL-248) epithelial cells were maintained in RPMI 1640 or Dulbecco’s modified Eagle’s medium (DMEM):Ham F12 (1:1) (Sigma-Aldrich, St. Louis, MO) supplemented with 10% heat-inactivated fetal bovine serum (FBS) and penicillin/streptomycin mixture (100 units penicillin and 0.1 mg streptomycin/mL; Sigma-Aldrich) at 37°C, 5% CO_2_ in saturated humidity. Cells were seeded onto 96 tissue-culture-treated well-plates (Thermo, United States) or 6 × 10^4^ cells/transwell (6.5-mm diameter and 3-μm pore size; Corning, NY) and cultured for about 10 days until transepithelial electrical resistance (TEER) reached 3000 Ω/cm^2^. TEER was measured using a Millicell ERS Volt-Ohm meter (MilliporeSigma, Burlington, MA). The culture medium was replaced with an antibiotic-free medium the day before the experiment.

### Adherence, invasion, and transmigration assay.

Polarized epithelial cells were incubated apically with bacteria (10^5^ CFU/well) and 0, 0.01, 0.1, or 1 μg/mL of AS101 at 37°C, 5% CO_2_ for 3 h to determine adherence. For invasion and transmigration assays, cells were incubated apically with bacteria (10^6^ CFU/well) alone at 37°C, 5% CO_2_ for 3 h, and 0, 0.01, 0.1, or 1 μg/mL AS101 was then added for another 3 h. The number of adherent GC was counted via spreading the 1% saponin-lysed cells onto the GCK agar plate. For the transmigration experiment, bacteria in the basolateral chamber were enumerated as the number of transmigrated GC. For invasion, infected cells were treated with gentamicin (200 μg/mL) for 2 h, washed with 1× PBS, and lysed with 1% saponin, and the resulting cell lysate was plated onto GCK agar.

### Inhibition of microcolony formation and growth.

GC was suspended in GC medium with Kellogg’s supplement and NaHCO_3_, the suspension was diluted to 10^7^ CFU/mL, and 200 μL GC was incubated in the presence or absence of AS101 in 8-well coverslip-bottomed chambers (Sigma-Aldrich) at 37°C, 5% CO_2_ for 4-h static incubation. For the growth inhibition test, 4-h preformed GC microcolonies were treated with 1 μg/mL of AS101 by direct addition or medium replacement and incubated for another 4 h with observation every 2 h. Images were acquired using a light microscope (ZEISS Axio Observer). Micrographs were analyzed using NIH ImageJ software to measure GC microcolony size in each aggregate.

### Transmission electron microscopy.

All specimens were prefixed in 2.5% glutaraldehyde/0.1 M sodium cacodylate buffer (pH 7.3) containing 1% tannic acid at 4°C overnight. After being washed in 0.1 M sodium cacodylate buffer with 5% sucrose, specimens were postfixed with 1% osmium tetroxide in 0.1 M sodium cacodylate buffer at room temperature for 2 h. Specimens were then washed in buffer, en-bloc-stained with 2% aqueous uranyl acetate, and dehydrated through a graded series of ethanol and twice with 100% acetone. Specimens were infiltrated and embedded with Spurr’s resin. Serial ultrathin sections of approximately 70 nm were cut with a diamond knife on a Leica Ultracut R ultramicrotome (Leica, Heerbrugg, Switzerland) and examined with a Hitachi H-7500 transmission electron microscope (Hitachi, Tokyo, Japan) at 80 kV. Images were acquired using an AMT NanoSprint12: 12 Megapixel sCMOS TEM Camera (Advanced Microscopy Techniques, Woburn, MA).

### ROS level.

A 2′,7′-dichlorodihydrofluorescein diacetate (DCFH-DA) ROS assay was utilized to detect the intracellular ROS levels of MS11 WT and MS11ΔOpa treated with AS101 at various concentrations as previously described (16). In brief, 10^9^ CFU/mL of bacterial strains was prepared in GC broth and incubated with DCFH-DA at 100 μM for 1 h at 37°C. The resulting cells were washed, resuspended, and adjusted to 10^7^ CFU/mL in GC broth. Subsequently, the bacterial suspensions were incubated with 1×, 2×, and 4× MIC of AS101 for 1 h at 37°C. The fluorescence signals were measured using a spectrofluorometric reader with an excitation wavelength of 500 nm and an emission wavelength of 530 nm. Viable bacteria were counted by serially diluting and plating the samples onto GC agar plates. The fluorescence intensities were normalized using the viable bacterial counts. All experiments were conducted in triplicate.

### Statistical analysis.

Statistical significance was assessed using the Student’s two-tail *t* test or one-way analysis of variance (ANOVA) depending on comparable properties. All the data were confirmed to fit into Gaussian distribution by Shapiro–Wilk test for normality. All analyses were performed using GraphPad Prism 8 software (GraphPad Software, San Diego, CA).
