# Supplementary material for: In Vitro Evaluation of Tellurium-Based AS101 Compound against Neisseria gonorrhoeae Infectivity
Source: Microbiol Spectr. 2023 Mar 6;11(2):e01496-22. doi: 10.1128/spectrum.01496-22 (PMC10100759; doi:10.1128/spectrum.01496-22)
Supplement: Supplemental file 1 — Supplemental material. Download spectrum.01496-22-s0001.pdf, PDF file, 0.5 MB [file spectrum.01496-22-s0001.pdf]

Fig. S1

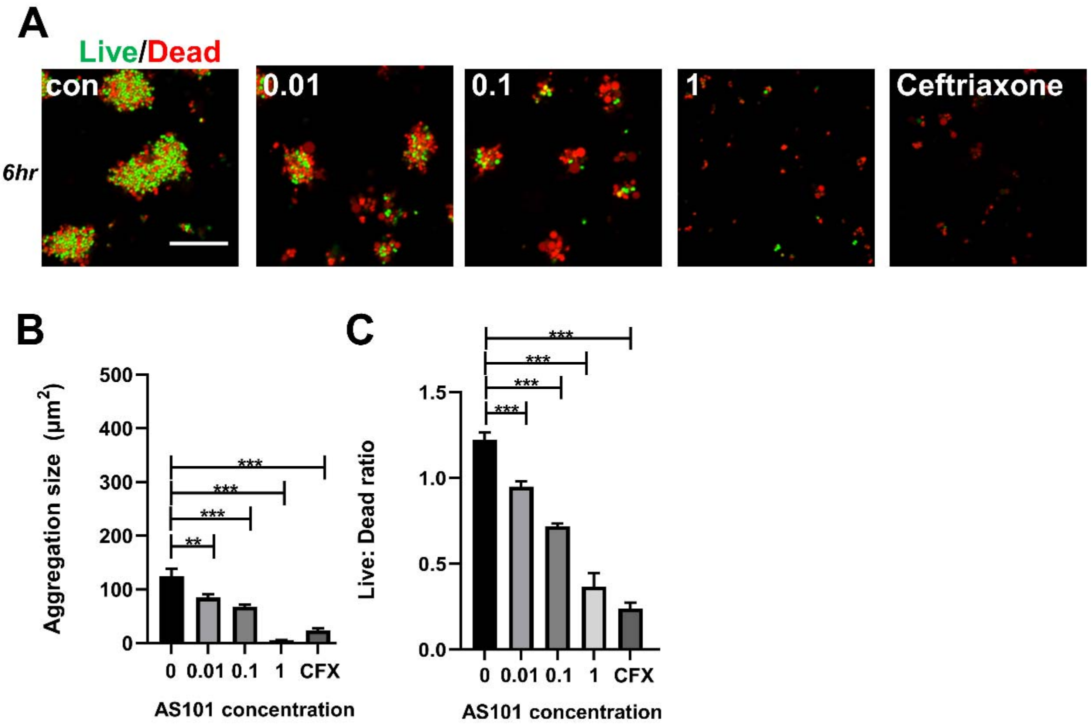

Fig S1. Live/dead bacteria distribution within aggregates under AS101 treatment. (A) 2x10^6 MS11ΔOpa was either incubated in the presence or absence of AS101 or ceftriaxone for 6 h. Aggregates were then stained to visualize viable (green) and dead (red) GC and visualized with confocal fluorescence microscope. CFX, Ceftriaxone treatment. Scale bar: 20 μm. The data were generated from eight randomly acquired fields from four independent experiments. Each data point indicates an individual aggregation. Images were then analyzed for (B) aggregation size and (C) ratio of live-to-dead GC. The bars represented the means of each group, and the lines on bars indicated the standard deviations. Statistical significance was determined using One-way ANOVA followed by Post-hoc Dunnett's test (***, $p<0.001$; **, $p<0.01$; *, $p<0.05$).

Fig. S2

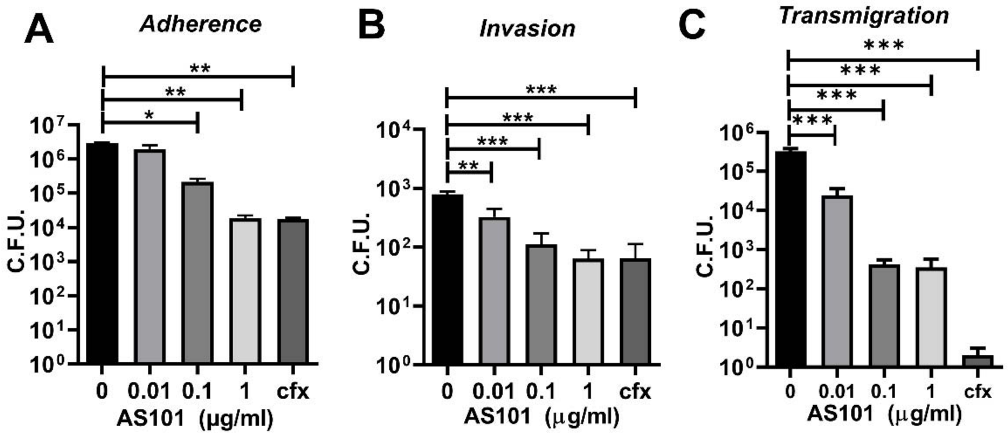

Fig S2. Effects of AS101 in GC infectivity in polarized epithelial monolayer. T84 cells were grown on transwell inserts and apically inoculated with MS11ΔOpa. (A) Various concentrations of AS101 was added apically along with GC inoculation and incubated for 3hr to determine the adherent GC. (B-C) Various concentrations of AS101 in media replaced the inoculated suspension after 3hr. (B) Invaded GC was quantified by the gentamicin protection assay. (C) Transmigrated GC was quantified by plating and counting the colonies from basal media. GC with 1 µg/ml of ceftriaxone (CFX) was served as a positive control. The bars represented the means of each group, and the lines on bars indicated the standard deviations. Statistical significance was determined using One-way ANOVA followed by Post-hoc Dunnett's test (***, $p<0.001$; **, $p<0.01$; *, $p<0.05$). All exepriements were conducted at least in triplicate.

Fig. S3

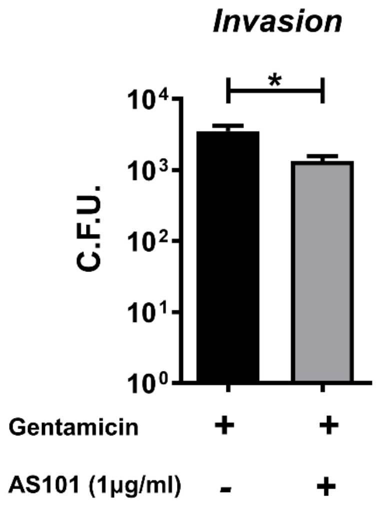

Fig S3. Effects of AS101 in invaded GC. ME180 cells were grown on transwell inserts and apically inoculated with MS11ΔOpa. After 6 hr incubation, gentamicin protection assay was performed in the absence or presence of AS101 (1 μg/ml) to determine anti-GC effect inside cells. Three independent experiments were performed. The bars represented the means of each group, and the lines on bars indicated the standard deviations. Statistical significance was determined using Student's T-test (*, $p<0.05$). All exepriements were conducted at least in triplicate.

Fig. S4

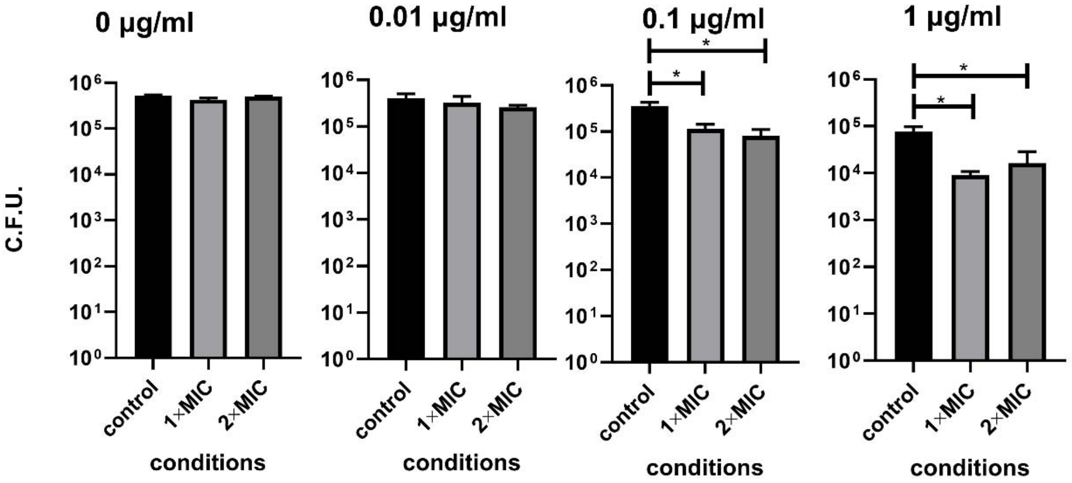

Fig S4. The effects of AS101 on azithromycin-resistant GC. Agar-dilution-based selection of azithromycin-resistant MS11WT was performed until isolates against 1X and 2X MIC of azithromycin were found. The isolates were inoculated into a 96-well plate and incubated in the presence or absence of AS101 for 6 h. The medium was collected and plated to determine the survival GC. Four independent experiments were performed. The bars represented the means of each group, and the lines on bars indicated the standard deviations. Statistical significance was determined using One-way ANOVA followed by Post-hoc Dunnett's test (*, $p<0.05$).

Fig. S5

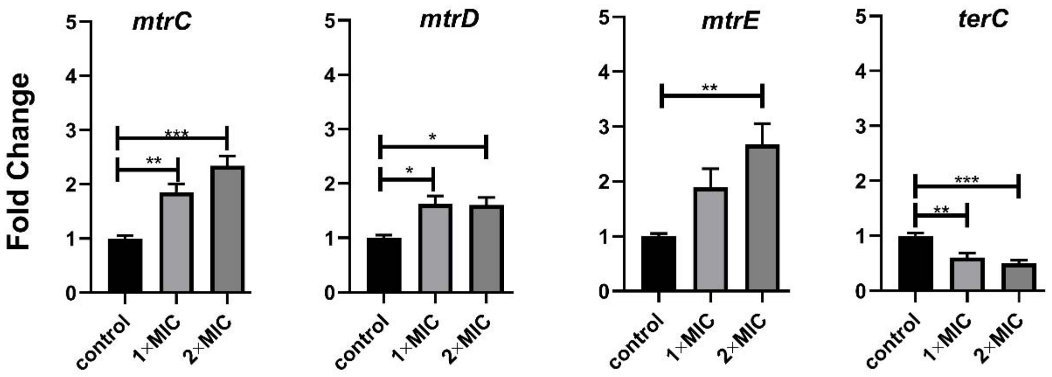

Fig S5. The MtrCDE and tellurium resistance gene expression in azithromycin resistant GC. The mRNA from MS11 WT and azithromycin resistant GC were extracted and followed by RT-qPCR for mRNA expression. The expression of *mtrC, D, or E,* and *terC* from azithromycin resistance GC were compared relative to its expression in MS11 WT control. Four independent experiments were performed. The bars represented the means of each group, and the lines on bars indicated the standard deviations. Statistical significance was determined using One-way ANOVA followed by Post-hoc Dunnett's test (***, p<0.001; **, p<0.01; *, p<0.05).

Concentration (μg/ml)	Mutation frequency (mean±SD)*10^{-3}			
	MS11		*WHO*	
	WT	Δopa	Y	Z
**0.05**	**9.625±2.446**	**7.75±3.536**	**12.29±3.352**	**7.625±1.598**
**0.1**	**7.375±1.061**	**7.625±1.408**	**14.14±1.069**	**1.875±0.296**
**0.4**	**0.531±0.168**	**0.5±0.185**	**4.426±1.134**	**0.037±0.007**

TableS1. Mutation frequency of AS101 treated GC strains. 10^3-10^6 C.F.U. of MS11 WT, MS11 ΔOpa, WHO Y, and WHO Z were grown on GCK agar with concentrations of AS101 from 0.001–1.0 μg/mL. Mutation frequency was determined by the ratio of viable GC count to the inoculated GC number.

Supplemented Material and Methods

Viable GC distribution in AS101-inhibited microcolonies

GC was suspended in GC media with Kellogg's supplement and $NaHCO_3$, the suspension was diluted to 10^7 CFU/mL, and 200 μL GC was incubated statically in the presence or absence of AS101 in 8-well coverslip-bottom chambers (Sigma-Aldrich, United States) at 37°C, 5% CO_2 for 6 h. Live/ Dead BacLight Stain (Life Technology, U.S.A) was used to stain the aggregates for 15 min, and images were acquired using a confocal microscope (Leica SP5X). Micrographs were analyzed using NIH ImageJ software to measure GC microcolony size and the fluorescence intensity ratio (FIR) of live to dead GC in each aggregate.

RNA extraction and reverse transcription

Total RNA samples of wild-type GC and the isolates with 1×, 2×, 4×, or 8× azithromycin MICs were extracted using TriPure Isolation Reagent (Roche, Mannheim, Germany) and the protocol from the previous study (1). Extracted RNA samples were stored at −80 °C. First-strand cDNA was synthesized from the total RNA using M-MLV Reverse transcriptase (Promega, United States) following the manufacturer's recommended protocol.

qRT-PCR

qRT-PCR was performed on a QuantStudio™ 6 Flex real-time PCR system (Bio-Rad, United States) using the GoTaq qPCR Master Mix (Promega, United States). The reactions were performed in a 10-μL volume mix containing 0.2-μL of the GoTaq qPCR Master Mix, 5 pmol/L specific primers, and approximately 2000 ng cDNA. The thermal cycling profile consisted of an initial denaturation at 95 °C for 2 min, followed by 40 cycles of 95 °C for 3 s, 60 °C for 30 s, and 16 °C for 30 s. The comparative Ct (ΔΔCt) method was used to evaluate the expression level of target genes (2). Basically, transcript abundance for each gene was normalized using the housekeeping gene pyruvate dehydrogenase (*pdhC*) as previously described (3). Each gene expression level was calculated by $2^{-\Delta\Delta Ct}$. All data were given in terms of relative mRNA expressed as means ± SEM. Five independent experiments with 2–3 technical replicates were performed. The Ct of each replicate was read three times for accuracy. The primer sequence used in above qRT-PCR were shown below.

Primer pair	Sequence (5'-3')	Target Gene
PdhC F<br>PdhC R	GTTCCGGTACGATTCTGCAAGAAG<br>CGGTTTCTTTGCTGACTTTGCCT	Pyruvate dehydrogenase
MtrC F<br>MtrC R	GATTGCGGTCGGCATCAA<br>GATCCATCAGCACACGCACATA	Multiple transferable resistance C
MtrD F<br>MtrD R	ATTTTGAACCTGACCGTGCC<br>CCCATTCGAAGCTGTAACCG	Multiple transferable resistance D
MtrE F<br>MtrE R	TCGAGCGCAATACCAGTTTG<br>GTCCGACATTGTAGCTGCTG	Multiple transferable resistance E
TerC F<br>TerC R	GTATTGCGCACCGTCATGAT<br>GAACGACTTTCTTGACGGCA	Tellurium resistance

References

1. Christodoulides M. 2019. *Neisseria gonorrhoeae*: methods and protocols. Springer.
2. Livak KJ, Schmittgen TD. 2001. Analysis of relative gene expression data using real-time quantitative PCR and the $2^{-\Delta\Delta CT}$ method. Methods 25:402-408.
3. Viscidi RP, Demma JC. 2003. Genetic diversity of *Neisseria gonorrhoeae* housekeeping genes. J Clin Microbiol 41:197-204.
